# Continuous flow synthesis of a pharmaceutical intermediate: a computational fluid dynamics approach†

**DOI:** 10.1039/c8re00252e

**Published:** 2019-01-30

**Authors:** Cameron T. Armstrong, Cailean Q. Pritchard, Daniel W. Cook, Mariam Ibrahim, Bimbisar K. Desai, Patrick J. Whitham, Brian J. Marquardt, Yizheng Chen, Jeremie T. Zoueu, Michael J. Bortner, Thomas D. Roper

**Affiliations:** a Chemical and Life Science Engineering, Virginia Commonwealth University, Richmond, VA, 23219 USA; b Department of Chemical Engineering and Macromolecules Innovation Institute, Virginia Polytechnic Institute and State University, Blacksburg, VA, 24061 USA. E-mail: mbortner@vt.edu; c MarqMetrix, Inc., Seattle, WA, 98103 USA

## Abstract

Continuous flow chemistry has the potential to greatly improve efficiency in the synthesis of active pharmaceutical ingredients (APIs); however, the optimization of these processes can be complicated by a large number of variables affecting reaction success. In this work, a screening design of experiments was used to compare computational fluid dynamics (CFD) simulations with experimental results. CFD simulations and experimental results both identified the reactor residence time and reactor temperature as the most significant factors affecting product yield for this reaction within the studied design space. A point-to-point comparison of the results showed absolute differences in product yield as low as 2.4% yield at low residence times and up to 19.1% yield at high residence times with strong correlation between predicted and experimental percent yields. CFD was found to underestimate the product yields at low residence times and overestimate at higher residence times. The correlation in predicted product yield and the agreement in identifying significant factors in reaction performance reveals the utility of CFD as a valuable tool in the design of continuous flow tube reactors with significantly reduced experimentation.

## 1 Introduction

Continuous flow chemistry is becoming prevalent as both academic and industrial researchers seek to improve upon traditional batch processes.^1–^^3^ In particular, the pharmaceutical industry has made strides to incorporate continuous flow methods to chemical synthesis and processing due to the potential for process intensification, as well as the ability to use higher temperatures and pressures than traditional pharmaceutical batch equipment may allow.^4–^^7^ These advantages lead to more efficient processes and the potential for improved process control and automation resulting in high performance in terms of product quality, consistency, waste production, and cost.^1–^^5,^^8,^^9^

In order to achieve these improved processes, it is critical that careful and thorough studies of reaction conditions are carried out.^10,^^11^ Fortunately, continuous flow chemistry lends itself to efficient experimentation as a result of its capability for changing multiple variables in sequence.^1–5^ For instance, the flow rate of a single reagent and the temperature of a reactor can be varied to collect data over a range of operating parameters without having to modify the experimental setup. Additionally, the ease of scalability of continuous flow chemistry allows for process development to be performed at lower scales before transitioning to a production scale, further reducing time and waste.^2^

Even with the use of continuous flow chemistry, experimental optimization can be tedious and potentially expensive if costly reagents must be used. This is particularly true when using the common one-variable-at-a-time (OVAT) approach.^11^ An alternative approach to OVAT-based experimental optimization is statistical design of experiments (DoE). DoE utilizes statistical modeling to identify the variables which have a significant effect on a chosen response. The implementation of fractional factorial design is particularly useful in screening studies as it reduces the number of experiments needed to identify those significant factors while elucidating interactions between the variables that the OVAT approach is unlikely to uncover.^10,^^11^ Even with the fractional factorial design, DoE requires significant experimentation, time and resources.

In contrast to experimental approaches, computational simulations allow for optimizing many variables without necessitating the consumption of chemical resources. The modeling of organic reactions can be challenging due to complex reaction profiles which can result in radial gradients of temperature and concentration complicating the use of commonly used flow reactor design equations.^12^ Nevertheless, the impact of these gradients can be determined through numerical methods such as computational fluid dynamics (CFD). These methods incorporate multiple coupled transport equations which govern the multiple reactant, diluent, and product species' behavior throughout the flow reactor.^13^ With the ever-increasing availability of computing power, CFD has been growing in popularity in recent years for simulating complex pharmaceutical synthesis reactions saving time and costs *versus* experimental based approaches.^14–^^17^

Both DoE and CFD are widely reported in the literature for process optimization;^11,^^14,^^18–^^20^ however, they have often been used tangentially, in that CFD is used to provide a possible theoretical justification for the experimental work.^17,^^21–^^23^ The aim of this work is to evaluate the capabilities of CFD for screening the effect of various experimental conditions on product yield. By comparison of CFD and an experimental DoE, the predictive capabilities of CFD are evaluated both in absolute prediction of product yields as well as the identification of the most significant experimental factors which impact the product yield.

**Fig. 1:**
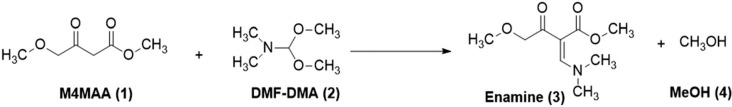
Reaction scheme studied for the synthesis of a DTG intermediate.^2^

For the current work, the comparison of results obtained for CFD and DoE was conducted for the reaction shown in Fig. 1. This reaction is the first step in the GlaxoSmithKline synthesis of cabotegravir^24^ as well as the recently reported flow synthesis of dolutegravir (DTG),^25^ an HIV integrase inhibitor that is recommended as part of a universal first line combination therapy for treatment of HIV-AIDS.^26,^^27^

## 2 Materials and methods

### 2.1 Materials

Methyl 4-methoxyacetoacetate (**M4MAA**), was procured from Oakwood Chemical (Estill, SC) and was distilled to greater than 99% purity before use. *N*, *N*-Dimethylformamide dimethylacetal (**DMF-DMA**, 97%) and HPLC-grade methanol (**MeOH**) were used as received from Thermo Fisher Scientific (Waltham, MA).

### 2.2 Experimental apparatus

**Fig. 2:**
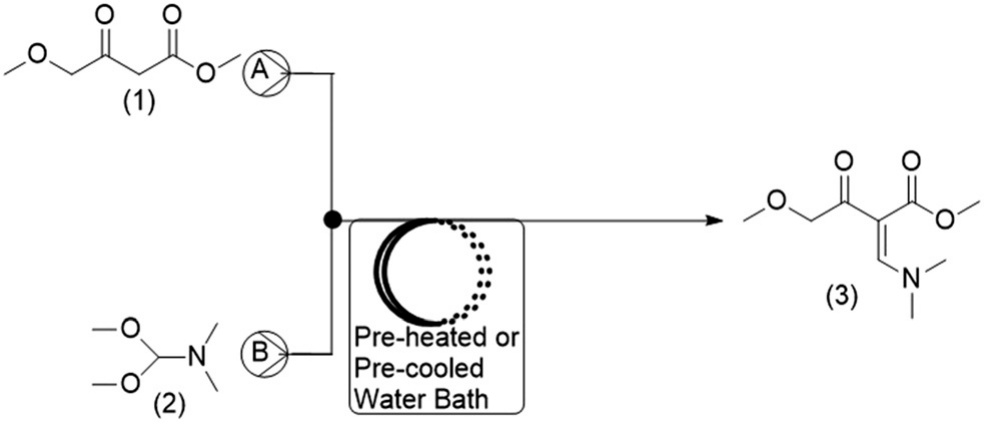
Experimental reactor setup.

A schematic of the reactor setup is shown in Fig. 2. Two Chemyx Nexus 3000 (Stafford, TX) syringe pumps were used along with 10 mL glass gas-tight syringes (SGE, Victoria, Australia) to pump reagents **M4MAA** (**1**) and **DMF-DMA** (**2**) to the tee junction and through the reactor coil respectively. The tubing reactors used in the flow experiments were constructed using 1.59 mm outer diameter (OD) PTFE tubing with either a 1.0 mm or 0.25 mm inner diameter (ID), polyetheretherketone (PEEK) tee junctions, and were assembled using suitable nuts and ferrules (IDEX Health and Science). The polytetrafluorethylene (PTFE) reactor coil was immersed in a water bath that was pre-heated or pre-cooled to the desired reaction temperatures.

### 2.3 Experimental reaction procedure

Sampling of crude reaction output was performed following flow reaction equilibration for three residence times, and subsequently quenched with toluene. The reaction mixture was analyzed *via* high-performance liquid chromatography (HPLC) with diode array detection (DAD) using an Agilent HP1100 system (Santa Clara, CA). HPLC peak areas for the starting material **M4MAA** (**1**), product **Enamine** (**3**), and a single impurity of unknown structure were calculated at 210 nm and corrected with relative response factors to calculate percent yield of product. Further details of the HPLC method and an example chromatogram are shown in the ESI† (Fig. S1).

### 2.4 Design of experiments

A screening fractional factorial design was performed to determine the significance of each variable in the continuous flow process and possible inter-factorial interactions. The variables studied were tubing length (*L*) and internal diameter (ID) of the reactor coil, volumetric flow rate (*Q*), temperature (*T*), and the initial molar ratio (*χ*) of **DMF-DMA** (**2**) to **M4MAA** (**1**). The upper and lower limits for each variable are depicted in Table 1 and were selected based off preliminary studies and commercially available tubing dimensions. A 2^5–^^1^ fractional factorial screening design (16 experiments) was chosen to maximize our understanding of the significant variables with a reduced number of experimental runs compared to a full factorial design (32 runs). Using a fractional factorial DoE design allows for the study of the five variables listed above without the need for every possible combination of variables to be run by dictating which specific variable combinations are required to statistically determine the effect of each individual variable. It should be noted that fractional factorial design will alias any three-way interaction terms with the two-way interaction terms;^10,^^28^ however, it was assumed that any three-way interaction terms would not be of a valuable significance compared to two-way and single variable effects.

**Table 1:** High and low variable values for fractional factorial design

Factor	High	Low
Length, *L* (m)	5	1
Inner diameter, ID (mm)	1	0. 25
Flow rate, *Q* (mL min^−1^)	1	0. 1
Temperature, *T* (°C)	40	10
Molar ratio, *χ*	1. 5	0. 95

Design-Expert software (Version 10, Stat-Ease, Minneapolis, MN) was utilized for the design and statistical analysis of the experiments. Each experiment was performed in triplicate (48 total runs) and completed in random order as specified by the design software. Center-points for this DoE could not be properly implemented due to a restraint on tubing ID; the true midpoint of 1.0 mm and 0.25 mm ID values is 0.625 mm which was not readily available.

CFD simulations were performed at the intended experimental DOE conditions so that direct comparisons could be made. While the factors and levels were identical, a full-factorial design was used for CFD simulations as the additional data points only required additional computation time and replicates were not needed.

For both the experimental and CFD DoE analyses, the percent yield of **Enamine** (**3**) was used as the response. The significant factors identified in the two DoE analyses were then compared to evaluate the applicability of CFD for optimization of reaction parameters.

### 2.5 Determination of empirical parameters

To accurately simulate the reaction conditions using CFD, empirical parameters must first be determined to describe reaction kinetics and defined material properties. For determination of kinetic parameters, *in situ* Raman spectroscopy was performed using a Marqmetrix (Seattle, WA) All-In-One Raman spectrometer equipped with a TouchRaman BallProbe to monitor batch reactions at various temperatures between 10 °C and 40 °C and molar ratios of 1.3 : 1 and 2 : 1 **DMF-DMA : M4MAA**. Multivariate curve resolution-alternating least squares (MCR-ALS) was used to extract reaction trends for each reactant.^29,^^30^ The reaction was found to follow second-order reaction kinetics (first order in both reactants) and an Arrhenius plot (depicted in Fig. S3†) was used to determine the activation energy (*E*_a_ = 57.93 kJ mol^−1^) and the pre-exponential factor (*A* = 2.00 × 10^8^ L mol^−1^ min^−1^). *In situ* infrared spectroscopy using a ReactIR 15 equipped with a DST series fiber conduit probe (Mettler Toledo, Columbus, OH) was used to validate the kinetic results.

The specific heat of each reaction component and the heat of reaction were experimentally determined using the EasyMax batch reactor and HfCal calorimetry unit (Mettler Toledo, Columbus, OH). The viscosities of each reaction component, listed in Table S2† were found using a Brookfield LVDV-E viscometer with a Brookfield SC4-18 spindle and 13R sample chamber. Samples were tested with 6.7 mL over a range of shear rates from 0.4 to 130 s^−1^.

### 2.6 Computational fluid dynamics

A reactor was simulated with COMSOL Multiphysics^®^ (Version 5.3, COMSOL, Inc., Burlington, MA) employing Chemical Reaction Engineering and Heat Transfer modules following the reactor design in Fig. 2. Table S1† highlights the governing equations incorporated for time-dependent systems. ^31^ The reactor mesh was generated with COMSOL's physics-based meshing algorithm with the mesh element size set to extremely fine. This method utilizes triangular elements to discretize the reactor followed by subsequent insertion of a rectangular boundary mesh to refine the elements describing the boundary layer gradients.^32^ A straight tube was constructed in lieu of a coil to reduce computational complexity, while assuming effects from curvature of the coil on velocity, concentration, and temperature were minimal due to a small element size, 0.4 mm, with respect to the radius of curvature of coil, 150 mm. A separate, three-dimensional simulation incorporating a tee junction was also developed to validate the assumption that mixing at the junction leading to the reactor occurs almost instantaneously with respect to residence time at the Péclet numbers of interest in this study. The mass, heat, and fluid transport equations were linearly discretized in all independent variables except the concentration of each species which were quadratic in their discretization. The equations were solved numerically using a fully coupled parallel sparse direct and multi-recursive iterative linear solver (PARDISO) using Newton's method with a constant damping factor of 0.9. Convergence was specified through achieving an absolute error estimate less than 5 × 10^−4^. Time stepping was implemented with a second order backwards differentiation formula and time steps were chosen freely by the solver. Four meshes were generated for each reactor geometry considered in our design, their respective number of mesh elements are outlined in Table 2. The simulations were verified to hold results independent of the number of mesh elements through solving the system with 2.5, 5, 10, 25, and 50% of the number of elements in our final simulation. Yield outputs remained constant at each mesh density therefore it was assumed sufficient meshing density was achieved in the simulations.

**Table 2:** Number of mesh elements for CFD simulations in each reactor geometry

Reactor length (m)	Reactor ID (mm)	Mesh elements
1	1	18 327
1	0.25	81 188
5	1	103 154
5	0.25	403 962

The simulations were carried out using a supercomputing cluster of 96 cores of Intel^®^ Xeon^®^ 2× E5-2680v3 @ 2.5 GHz CPU with 128GB RAM @ 2133 MHz and were completed in 360 CPU hours (Advanced Research Computing, Blacksburg, VA). Product yields were then determined by the ratio of **Enamine** (**3**) to the limiting reagent at the output of the reactor.

## 3 Theory

### 3.1 Governing equations

The reaction species were found to behave as Newtonian fluids within the shear rate range applicable to our reactor, 10–160 s^–1^. The fluid density was found to be a function of the extent of reaction and varied with concentration spatially within the reactor. Calculated Reynolds numbers ranged from 0.9–4.9 for tube IDs of 0.25 mm and 1.0 mm respectively. As such the fluid was treated as a compressible Newtonian fluid in laminar flow with the Navier–Stokes and continuity equations. Heat transport is considered through conservation of energy as well as chemical species transport which is governed by Fick's law with the reaction rate expression in Table S1.† The governing equations implemented in this work, along with fluid and species properties, are in Tables S1 and S2,† respectively.

### 3.2 Mixture relationships

Equations for the evaluation of mixture properties were implemented for the density, viscosity, and heat capacity of the fluid.

$$\bar{\rho }=\frac{1}{\sum_{i}\frac{w_{i}}{\rho_{i}}}$$ (1)

where *w*_i_ and *ρ*_i_ are the density and mass fraction of each species respectively. The ideal solution viscosity is given by

$$\bar{\mu }=e^{{\sum_{i}n}_{i}\ln\;\mu_{i}}$$ (2)

where *n*_i_ and *μ*_i_ are the mole fraction and viscosity of each individual species, respectively. The mass-averaged heat capacity is given by

$${\bar{C}}_{p}=\sum_{i}\frac{c_{p,i}w_{i}}{M_{i}}$$ (3)

where *C*_p,i_ and *M*_i_ are the molar heat capacity and molar mass of each species, respectively.

### 3.3 Boundary and initial conditions

In the CFD simulation, the reactor was initially filled with a concentration gradient of **M4MAA** (**1**) over the reactor length which approached zero at the reactor outlet providing a more appropriate starting point than an initially empty reactor state. Time-dependent simulations were carried out from time *t* = 0 to a final time which was defined as five times the residence time for each respective reactor design. This ensured the system reached steady state and any effect of the initial reactant concentrations at time zero was removed. A symmetry constraint, $\frac{\partial f}{\partial r}{|}_{r=0}=0$, was applied to simulate the axial symmetry of the reactor, where *f* is each respective transport variable (*T*, *u*, and *N*) and *r* is the radial spatial coordinate. The outside walls of the tubing in the simulation were set to the desired reactor temperature and kept constant over the course of the reaction. This mimics the water bath used in the experimental reactor design.

## 4 Results and discussion

### 4.1 Computational fluid dynamic simulation

Reynolds and Péclet numbers were calculated to evaluate the impact of convection and diffusion on the mixing of the two reactants at the tee junction. The pairwise binary diffusion coefficients were not known for all combinations of our chemical species so the software default isotropic diffusion coefficient of 1 × 10^−9^ m^2^ s^−1^ was implemented. A sensitivity analysis was performed on the diffusion coefficients used in the CFD simulations to evaluate the validity of this assumption using a simulation of the reaction condition most susceptible to a change in diffusivity. Thus, the simulated reaction with the longest residence time (39.3 min), smallest molar ratio (0.95 : 1 **DMF-DMA : M4MAA**), and greatest temperature (40 °C) was subjected to a sweep of diffusivities ranging from 1 × 10^−6^ to 1 × 10^−12^ m^2^ s^−1^. As shown in Fig. S10,† product yield is independent of diffusion coefficient signifying that the system is not limited by mass transfer effects and is instead kinetically limited.

**Fig. 3:**
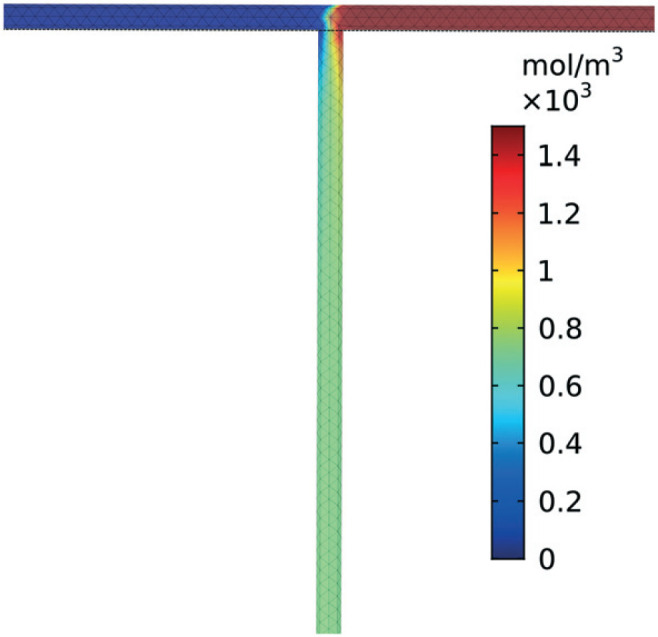
Three dimensional CFD mixing, without reaction, of 1.5 mol m^−3^ of both **M4MAA** (**1**) and **DMF-DMA** (**2**) entering a tee junction from left and right respectively and exit mixed downward. Pé and Re numbers for this simulation are 1.51 × 10^5^ and 5.02 respectively. Red and blue indicate high and low concentrations of **DMF-DMA** (2) respectively. The dashed line indicates where the reaction was assumed to initiate due to removal of the tee junction.

Low Reynolds numbers, on the order of unity, are observed within our simulated reactor signifying laminar flow throughout the straight channels. High Péclet numbers, 2.8 × 10^3^–3.5 × 10^5^, at the tee junction show that convection dominates significantly over diffusive transport regardless of the relatively slow flow rates. The combination of high Péclet number and low Reynolds numbers has been shown to exhibit stable chaotic advection arising from the sharp 90° turn in the tee.^33^ As a result, convective transport remains dominant over diffusive transport validating our assumption that mixing completes quickly after the tee junction. The three-dimensional simulation, Fig. 3, further confirms that the fluid was mixed approximately 2 cm after the tee. A comparison of reactors with and without this tee junction result in minimal deviation in product yield (<0.1%). Therefore, we neglected the tee junction in further simulations and assume completely and instantaneously mixed reactant flow through the inlet of the reactor.

At the inlet of the reactor, a radial and axial temperature gradient was observed due to the introduction of room temperature reactants into either a cooled or heated water bath per the design conditions of our experiments. These gradients dissipated after approximately 0.2 m and 0.5 m from the reactor inlet corresponding with simulations with low and high fluid velocity. At this point an isothermal reactor state was seen throughout the remainder of the reactor. The exothermic nature of our reaction had a meaningful impact on the heat transfer profile of our reactor near the inlet, however, the reduced reaction rate in the second half of the reactor led to a decrease in its significance.

**Fig. 4:**
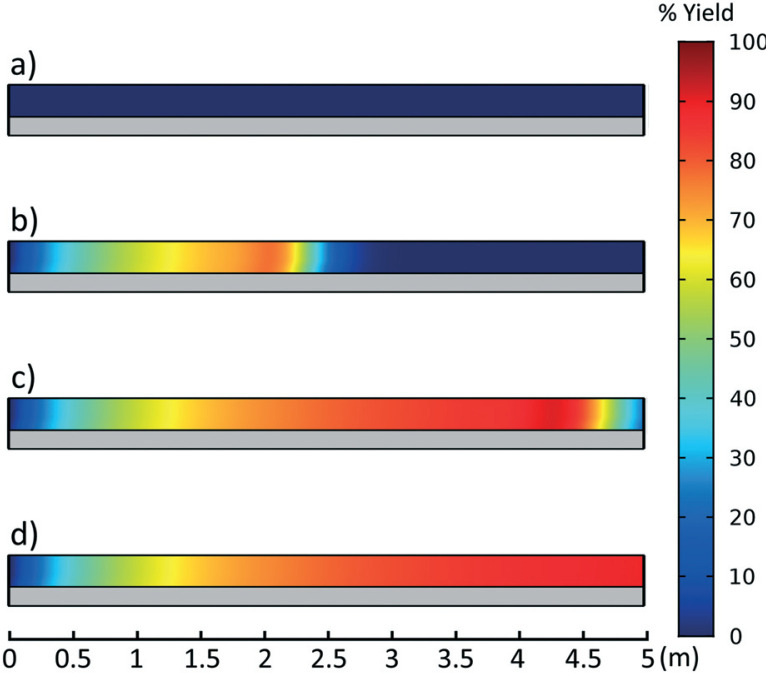
Percent yield as a function of reactor length at a) 0, b) 0.5, c) 1.0, d) 5.0 times the residence time. Reactor length 5 m, 1 mm ID, 40 °C, 0.1 ml min^−1^ flow rate, and 0.95 : 1 molar ratio. Reactor inlet at left and exiting right. Reactor dimensions not to scale.

Product yields were calculated as the ratio of **Enamine** (**3**) to the limiting reagent (as dictated by stoichiometry) at the output of the reactor at steady state. Transient data, Fig. 4, was also collected to observe the startup behavior of the reactor. It was found that a steady state solution was obtained after 2.5 residence times for all reaction conditions.

**Fig. 5:**
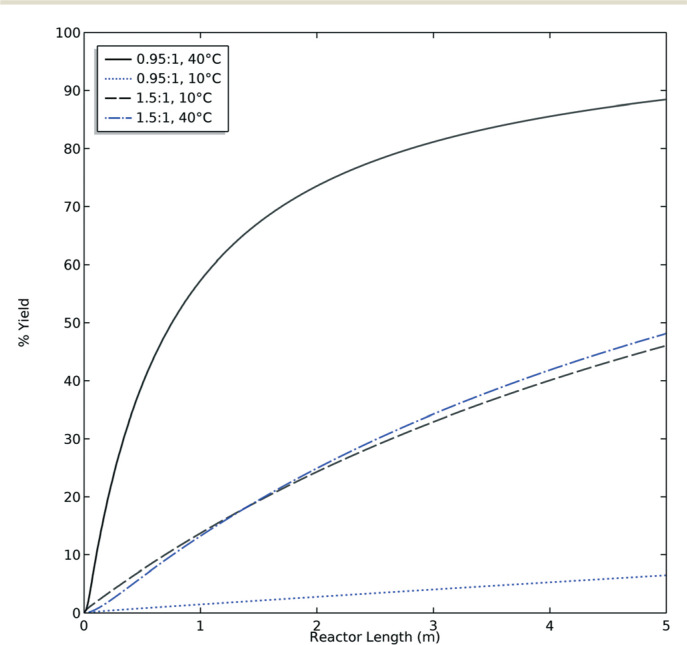
Theoretical product yield with respect to the limiting reagent as a function of reactor length at both high and low values of molar ratio and temperature predicted by CFD. Black and blue lines represent and 0.1 and 1.0 ml min^−1^ flow rates, respectively. Reaction condition: 5 m reactor with 1 mm ID.

This CFD simulation was utilized to simulate a full factorial DoE to identify which reaction parameters significantly impact product yield. Representative results are shown in Fig. 5 to highlight the effects of molar ratio and temperature over the length of the reactor. Full results are shown in Fig. S6–S9.† Increasing temperature and molar ratio of **DMF-DMA : M4MAA** was found to increase product yield through an increase in reaction rate.

### 4.2 DoE analysis

**Fig. 6:**
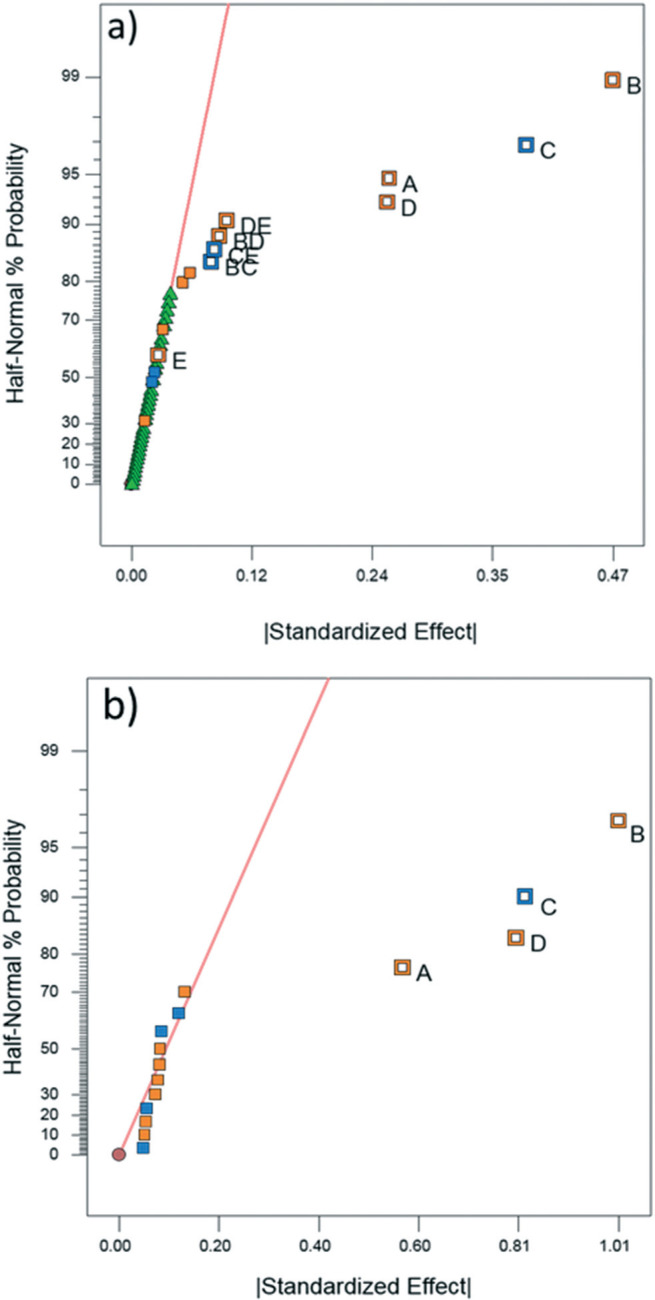
Half-normal percent probability plot for (a) experimental product yield and (b) CFD predicted product yield. Factors are labeled as follows: A) tubing length B) inner diameter C) flow rate D) temperature and E) molar ratio. Orange and blue points represent factors which are positively and inversely correlated with yield respectively.

DoE analyses were performed for both CFD and experimental results in order to screen the variables that significantly affect the product yield. Significant factors were determined using the half-normal plots shown in Fig. 6 as well as Pareto plots shown in Fig. S11 and S12.†^10,^^11,^^28^ The relevant statistical metrics for both DoE analyses are listed in Table 3.

**Table 3:** Selected ANOVA statistics for experimental and CFD models

Statistical term	Experimental	CFD
*p*-Value (*F*-test, *α* = 0.05)	<0.0001	<0.0001
*F*-Value (*α* = 0.05)	56	96
*p*-Value (lack-of-fit test, *α* = 0.05)	0.27	
Lack-of-fit *F*-value (*α* = 0.05)	1.4	
Pure error	0.012	
*R*^2^	0.93	0.97
Adj. *R*^2^	0.91	0.96
Pred. *R*^2^	0.89	0.94

The DoE models containing the statistically significant factors for the experimental analysis and CFD simulations are shown in eqn (4) and (5), respectively. The relative importance of each factor can be deduced by the magnitude and sign of each coefficient. Considering that these are screening studies, the comparison between CFD and experimental model coefficients is limited to an order of magnitude analysis.

$$Log_{10}\left(\%yield_{ena\min e}^{EXP.}\right)=0.13L+0.25ID-0.19Q+0.13T+0.0013\chi -0.039ID\times Q+0.043ID\times T-0.041Q\times \chi +0.047T\times \chi +1.09$$ (4)

$$Log_{10}\left(\%yield_{ena\min e}^{CFD}\right)=0.29L+0.50ID-0.41Q+0.40T+0.78$$ (5)

ANOVA for both the experimental and CFD models indicates that both are significant with no significant lack-of-fit (*F*-value ∼1) for the experimental model.^10^ Lack-of-fit was not calculated for CFD due to a lack of replicates since repeated simulations would result in identical values. The experimental and CFD models also have *R*^2^ and adjusted *R*^2^ values that are in good agreement (difference less than 0.2) as well as high predicted *R*^2^ values, indicating that the models are appropriate.^10^ The experimental and CFD model coefficients and their respective *p*-values are listed in Table 3. Both CFD and experimental outputs identified tubing length, tubing ID, flow rate and temperature to be significant. Of the four single-factor terms, length, ID, and flow rate are components of residence time. The magnitude by which each term affects residence time is reflected in the coefficient of each factor in Table 4. ID is related to reactor volume, and thus residence time, through a squared term. Flow rate is inversely related to residence time and tubing length is directly proportional to residence time as a multiplicative term. According to the DoE model, increasing ID and reactor length (*i.e*., increasing residence time) increases product yield. Thus, the residence time is the most impactful factor to product yield, along with temperature. Temperature is the fourth single-factor term, owing to faster reaction kinetics at higher temperatures. The disparity in the significance of temperature and reactor length between the two models may be explained by the CFD simulation boundary condition depicting perfect heating throughout the reactor, whereas in the experimental setup it is likely that temperature gradients and imperfect heating occurred within the water bath and reactor coil, reducing the significance of temperature.

**Table 4:** Significant DoE model terms, coefficients and their *p*-values, and intercept

Experimental model			CFD model	
Factor	Coefficients*^a^*	*p*-Value prob. > *F*	Coefficients*^b^*	*p*-Value prob. > *F*
A-length (*L*)	0.13	<0.0001	0.29	<0.0001
B-inner diameter	0.24	<0.0001	0.50	<0.0001
(ID)				
C-flow rate (*Q*)	−0.19	<0.0001	−0.41	<0.0001
D-temp (*T*)	0.13	<0.0001	0.40	<0.0001
E-molar ratio (*χ*)	0.013	0.41*^b^*		
BC	−0.039	0.020		
BD	0.043	0.011		
CE	−0.041	0.016		
DE	0.047	0.0061		
Intercept	1.09		0.78	

a All coefficients are log-scaled.

b Included to maintain hierarchy.

**Fig.7:**
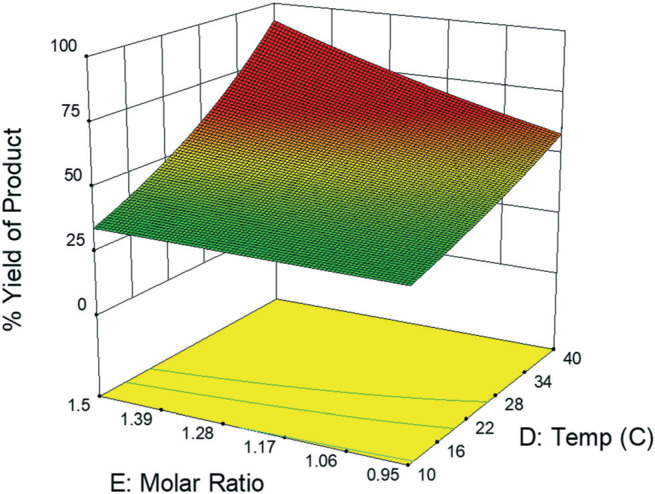
Surface plot of experimental data fractional factorial DoE model depicting percent yield of product as a function of molar ratio (*E*) and temperature (*D*) within their high/low value range where all other variables (*A, B, C*) are held constant.

The experimental DoE identified four statistically significant (*α* < 0.05) interaction terms while the CFD results did not detect any significant inter-factorial interaction. Reactant molar ratio was found to be insignificant as a single factor in the experimental DoE model; however, it is included because of its interaction with both temperature and flow rate. Fig. 7 represents a surface and contour plot of the interaction between molar ratio and temperature. At lower temperatures, molar ratio has no effect on product yield; however, at higher temperatures product yield is influenced by molar ratio. Thus, this interaction term was included in the experimental model to accurately predict the product yield.

### 4.3 CFD and experimental yield comparison

The accuracy of the yield predicted by CFD simulations was compared to the experimental yield. When analyzing the HPLC data for **M4MAA** (**1**) and **Enamine** (**3**), an unknown compound was consistently identified (Fig. S1†). Attempts to fully characterize this impurity were not successful and thus the mechanism of its formation could not be deduced; however, a small amount was isolated which allowed for a response rate to be calculated and thus the yield with respect to the **M4MAA** (**1**) was calculated for each reaction. The abundance of the impurity was less than 0.5% for most experimental runs but grew to 2–9% for runs with residence times over 3 minutes and at 40 °C. The rate of formation of this impurity was not able to be determined thus introducing uncertainty into the computational model and subsequent product yield figures.

**Fig. 8:**
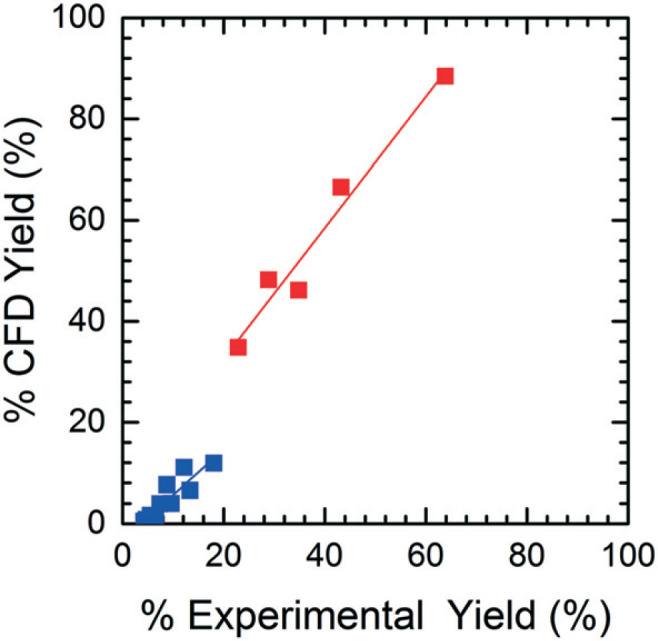
Prediction accuracy of the CFD simulations. Two distinct linear trends indicate overestimation at higher yields and underestimation at lower yields as indicated by the slopes of greater than and less than one, respectively.

Fig. 8 illustrates the CFD predicted **Enamine** (**3**) yield *versus* the experimental yield. This prediction plot is best interpreted as two distinct linear trends, where the lower yield values show a slope of 0.85 and the higher yield values give a slope of 1.31. The critical point between underestimation and overestimation occurs at approximately 20% yield.

**Fig. 9:**
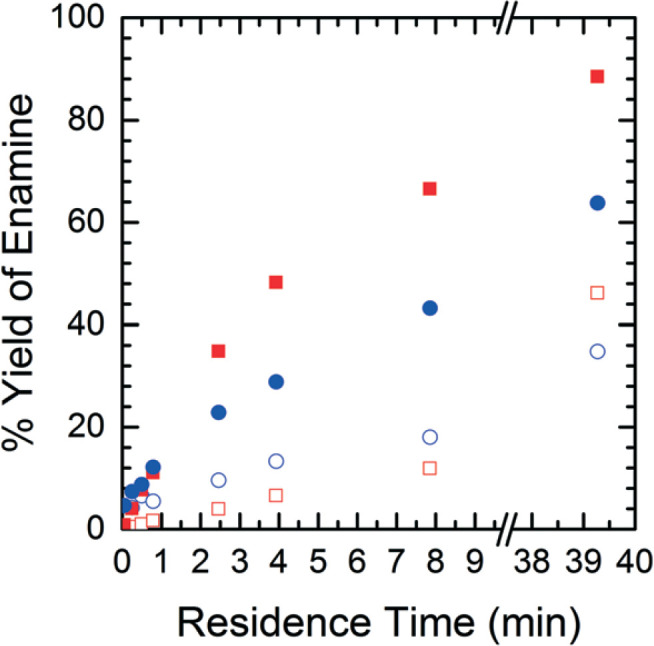
Product yield as a function of residence time – CFD simulated data (■), experimental data (●), solid symbols = runs at 40 °C, open symbols = runs at 10 °C

In order to understand the nature of these prediction errors, the percent yield of **Enamine** (**3**) along with the corresponding CFD simulation results with respect to residence time are shown in Fig. 9. As expected, increased temperatures and longer residence times lead to higher yields and higher absolute prediction errors. These errors were as low as 2.4% yield at low residence times (<5 min) and up to 19.1% yield at high residence times (>8 min). The crossover from under to overestimation is again observed to take place at approximately 20%. The abundance of the unknown impurity was also found to be the greatest (≥2%) at these longer residence times, suggesting that the increase in impurity concentration contributes to the deviation between the simulation and experimental results.

## 5 Conclusion

In this work the ability of CFD as an early stage tool for reactor design and reaction profiling in continuous flow chemistry, represented by the first step in the synthesis of dolutegravir, was demonstrated. The time savings of using CFD were exemplified in taking approximately one-fifth of the time to model the system compared to running the experiments. The agreement between the experimental and computational DoE analyses, in terms of the factors with a significant impact on the product yield, shows the potential of using CFD in the early screening studies to reduce experimental analysis and enable a more in depth screening of a wider range of variables. While the CFD analysis did not detect inter-factorial interactions, the contribution of those interaction terms to the experimental model, as measured by the model coefficients, were minor compared to the single-factor terms. A direct, point-by-point comparison also shows excellent correlation between results with absolute errors lower than 20% for all cases and lower than 10% in most cases. The division between over and underestimation of product yield may be influenced by the unidentified impurity detected in the experimental results. This signifies the importance of understanding the rate of formation of impurities to increase the accuracy of CFD reaction models. For practical application to flow system design, CFD is appropriate to provide an approximate product yield, which can be fine-tuned and optimized based on further experimentation that is guided by the significant factors and ranges identified in the CFD simulations. Furthermore, while this model was for a kinetically limited, single phase, non-selective reaction, appropriate assumptions and additional parameter specifications would enable a researcher to properly adapt this model to suit their specific needs. This strategy will improve efficiency of pharmaceutical process optimization both in terms of time and resources by combining a kinetics and computational approach, which generates significant process understanding, with a subsequent experimental approach to refine and develop a comprehensive reaction model.
