# Supplementary material for: Continuous flow synthesis of a pharmaceutical intermediate: a computational fluid dynamics approach
Source: React Chem Eng. 2019 Jan 30;4(3):634–42. doi: 10.1039/c8re00252e (PMC7745113; doi:10.1039/c8re00252e)
Supplement: Supplementary file 1 [file RCE-04-03-634-s001.pdf]

Electronic Supplementary Material (ESI) for Reaction Chemistry & Engineering.
This journal is © The Royal Society of Chemistry 2019

Supporting Information for

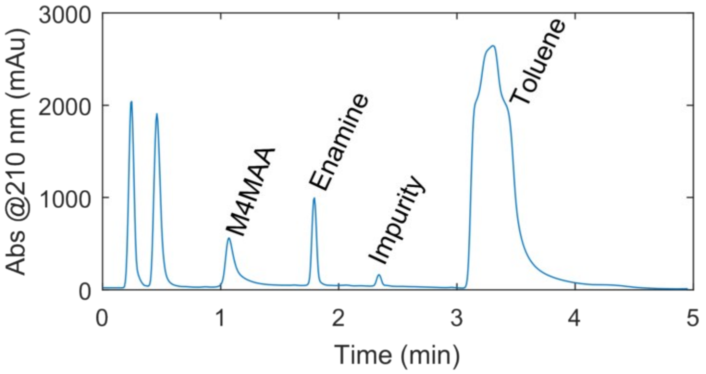

Continuous flow synthesis of a pharmaceutical intermediate: A computational fluid dynamics approach

Cameron T. Armstrong[1*], Cailean Q. Pritchard[2*], Daniel W. Cook[1], Mariam Ibrahim[1], Bimbisar K. Desai[1], Patrick J. Whitham [3], Brian J. Marquardt[3], Yizheng Chen[1], Jeremie T. Zoueu,[2] Michael J. Bortner[2], Thomas D. Roper[1]

1) Chemical and Life Science Engineering, Virginia Commonwealth University, Richmond, VA 23219
2) Department of Chemical Engineering, Virginia Polytechnic Institute and State University, Blacksburg, VA 24061
3) MarqMetrix, Inc., Seattle, WA 98103

* These authors contributed equally to this work

Analytical Methods

An Agilent 1100 HPLC with a diode array detector was utilized for determining yield and conversion of the reactions. A small aliquot from the output of the flow reaction was diluted in toluene to quench any remaining reaction and to dilute for HPLC analysis. HPLC was performed using a Gemini C18 column (50 x 3 mm, 5 µm; Phenomenex, Torrance, CA). A mobile phase gradient was utilized using 0.1% formic acid in water (A) and acetonitrile (B). Over 5 minutes %B was raised from 5% to 95%. A representative chromatogram is shown in Figure S1. The peak areas of **M4MAA**, **Enamine**, and a single impurity were determined at 210 nm and corrected using mass-based relative response factors. These corrected peak areas were used to calculate percent abundance by mass and converted to percent yield of the product as well as percent conversion of **M4MAA**.

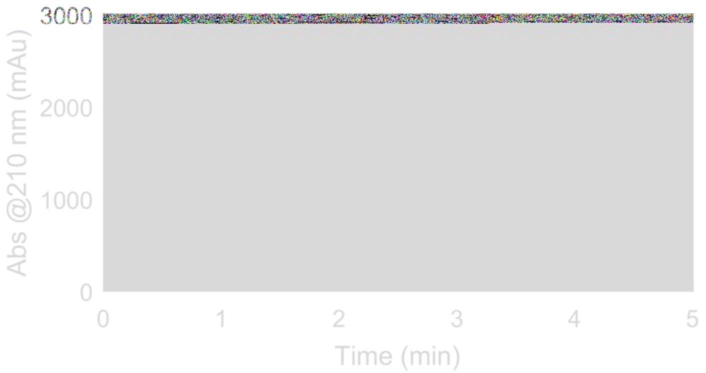

Figure S1. Representative chromatogram for the analysis of experimental reactions in flow.

Determination of Kinetic Parameters

Kinetic parameters were determined in batch reactions via *in situ* Raman spectroscopy and validated with *in situ* infrared (IR) spectroscopy. Reactions were performed in an EasyMax workstation (Mettler Toledo, Columbus, OH) using a 25 mL reactor with stirring at 700 RPM and at various temperatures. Raman spectra were collected using a MarqMetrix (Seattle, WA) All-In-One Raman spectrometer equipped with an immersion ballprobe. Solvent-free batch reactions were performed at molar ratios of 2:1 and 1.3:1 (**DMF-DMA**:**M4MAA**) with temperatures of 10 °C, 20 °C, 30 °C, and 40 °C.

Kinetic profiles were extracted from the raw data utilizing multiexperiment multivariate curve resolution-alternating least squares (MCR-ALS) to resolve individual chemical signals. All curve resolution was performed in the MATLAB programming environment (Mathworks, Natick, MA) using previously written in-house programs. For a description of the MCR-ALS method the reader is referred to previous literature.[1,2] All Raman experiments along with calibration samples for **DMF-DMA** and **M4MAA** were analyzed simultaneously via concatenation of the individual data matrices. Five components were used to represent the two starters, two products, and a fluorescent background which evolves over the course of the reaction. MCR-ALS was constrained with non-negativity in all components in both the kinetic and spectral modes. **DMF-DMA**, **M4MAA**, and **MeOH** reference spectra were constrained to exactly match reference spectra collected from neat samples. Sample selectivity was used on the calibration samples to reflect *a priori* knowledge of the composition of these samples.

The resolved kinetic profiles were converted to concentration curves via the calibration data. The data were plotted according to the integrated rate law (Equation S1; Figure S2) to calculate the rate constant (*k*).

$$\ln\left(\frac{[B][A]_0}{[B]_0[A]}\right) = k([B]_0 - [A]_o)t \quad \text{(S1)}$$

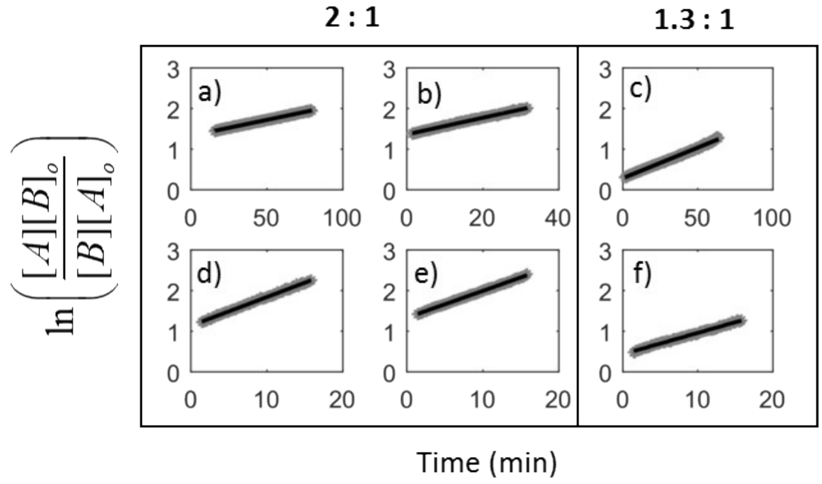

Figure S2. Integrated rate plot for the Raman kinetic experiments at 2:1 and 1.3:1 molar ratios (**DMF-DMA**:**M4MAA**) for temperatures a) 10 °C, b-c) 20 °C, d) 30 °C, e-f) 40 °C.

Activation energy (E_a) and the pre-exponential factor (*A*) were then found to be 57.93 kJ*mol^{-1} and 2.00 x 10^8, respectively, via the Arrhenius plot shown in Figure S3.

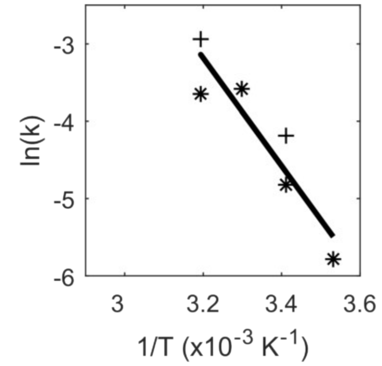

Figure S3. Arrhenius plot for the Raman kinetics experiments. Markers represents 2:1 (*) and 1.3:1 (+) mole ratio experiments. Bold line represents linear regression fit of data to determine activation energy from Arrhenius analysis ($y = -6969x + 19.12$; R^2 = 0.86).

These kinetic parameters were validated via IR spectroscopy. Experiments at a 1:1 molar ratio and multiple temperatures were monitored with a ReactIR (Mettler Toledo) with an immersion probe. Experiments were performed in an EasyMax workstation with a 100 mL reactor. Conversion to concentration profiles was performed with calibration curves based on peak height

for **DMF-DMA** and **M4MAA**. The pseudo-second order integrated rate law was used to calculate *k* for each experiment followed by the calculation of E_a and *A* (57.85 kJ*mol^{-1} and 1.71 x 10^8, respectively) via an Arrhenius plot (Figure S4) as described above. These values validated those calculated by Raman spectroscopy and thus the Raman kinetic parameters were used as inputs for the CFD model.

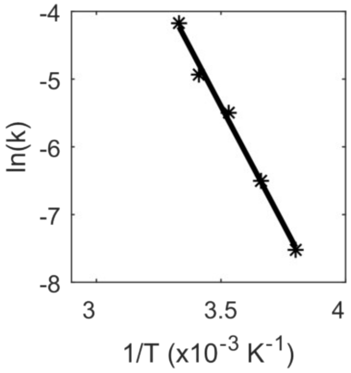

Figure S4. Arrhenius plot for the IR validation of kinetic parameters at 1:1 mole ratio. Bold line represents linear regression fit of data to determine activation energy from Arrhenius analysis ($y = -6958x + 18.95$; R^2 = 0.99).

Experimental Molar Ratio Control

The initial molar ratio for the reaction was accounted for by setting specific molar flow rates for **M4MAA** and **DMF-DMA**. These were implemented as independent volumetric flow rates on the syringe pumps for each reagent considering their molecular weights *MW*, densities ρ, and the molar ratio χ.

$$Volumetric\ Flow\ Ratio = \chi \cdot \frac{MW_{DMFDMA}}{\rho_{DMFDMA}} \cdot \frac{\rho_{M4M}}{MW_{M4M}} \quad \text{(S2)}$$

$$Q_{M4M} = \frac{Q_{total}}{(1 + Volumetric\ Flow\ Ratio)} \quad \text{(S3)}$$

$$Q_{DMFDMA} = Volumetric\ Flow\ Ratio \cdot Q_{M4M} \quad \text{(S4)}$$

Computational Fluid Dynamic Model Development

Table S1 CFD model governing transport equations and mixture rules

Governing Equations	
Continuity equation	$\frac{\partial \rho}{\partial t} + \nabla \cdot (\rho u) = 0$
Navier-Stokes	$\rho\frac{\partial u}{\partial t} + \rho(u \cdot \nabla)u = \nabla \cdot [-pI + \mu(\nabla u + (\nabla u$

Heat transport	$\rho C_p\left(\frac{\partial T}{\partial t} + u \cdot \nabla T\right) + \nabla \cdot q = Q$
Mass transport	$\frac{\partial c_i}{\partial t} + \nabla \cdot (-D\nabla c_i) + u \cdot \nabla c_i = R_i$
**Reaction Equations**	
Rate equation	$R_i = \nu_i k C_a C_b$
Arrhenius equation	$k = Ae^{-\frac{E_a}{RT}}$
**Mixture relationships**	
Mass-averaged density	$\bar{\rho} = \sum_i (\rho_i w_i)$
Ideal solution viscosity	$\bar{\mu} = e^{\sum_i w_i ln\mu_i}$
Mass-averaged heat capacity	$\bar{C}_p = \sum_i (C_{p,i} w_i)$

Table S2. Fluid and chemical species properties for CFD model

Symbol	Name	Value	Units
ρ_a	Density of pure species **M4MAA**	1129	$\frac{kg}{m^3}$
ρ_b	Density of pure species **DMF-DMA**	897	$\frac{kg}{m^3}$
ρ_c	Density of pure species **ENAMINE**	1000	$\frac{kg}{m^3}$
ρ_d	Density of pure species **MeOH**	792	$\frac{kg}{m^3}$
ρ_{PTFE}	Density of PTFE[3]	2154	$\frac{kg}{m^3}$
M_a	Molecular weight of species **M4MAA**	146.06	$\frac{g}{mol}$
M_b	Molecular weight of species **DMF-DMA**	119.09	$\frac{g}{mol}$
M_c	Molecular weight of species **ENAMINE**	201.10	$\frac{g}{mol}$
M_d	Molecular weight of species **MeOH**	32.01	$\frac{g}{mol}$
$C_{p,a}$	Heat Capacity of species **M4MAA**	485.6	$\frac{J}{mol \cdot K}$

$C_{p,b}$	Heat Capacity of species **DMF-DMA**	198.7	$\frac{J}{mol \cdot K}$
$C_{p,c}$	Heat Capacity of species **ENAMINE**	337.1	$\frac{J}{mol \cdot K}$
$C_{p,d}$	Heat Capacity of species **MeOH**	79.5	$\frac{J}{mol \cdot K}$
$C_{p,PTFE}$	Specific Heat Capacity of PTFE[4]	1000	$\frac{J}{kg \cdot K}$
ΔH_{rxn}	Heat of reaction	– 3.86	$\frac{kJ}{mol}$
E_a	Reaction Activation Energy	57.9	$\frac{kJ}{mol}$
A	Arrhenius Pre-exponential Factor	$3.34\ x\ 10^3$	$\frac{m^3}{s \cdot mol}$
$c_{a,pure}$	Concentration of purchased species **M4MAA**	7.73	$kmol/m^3$
$c_{b,pure}$	Concentration of purchased species **DMF-DMA**	7.53	$kmol/m^3$
k_{PTFE}	Thermal conductivity of PTFE[3]	0.272	$\frac{W}{m\,K}$

Mesh Independence

Finite element simulations were run with four different meshes in accordance with each geometric factor outlined in our DoE and shown in Table 2. All simulations for each DoE condition were performed in a series of meshing densities to verify the results are independent of the number of elements in our mesh. Each mesh was generated with approximately 2.5, 5, 10, 25, and 50 % of the number of elements in the final simulation, given in Table 2. A selection of this process for representative DoE trials for a 1 m long reactor with a 1 mm ID is illustrated in Figure S5 where product yield was analyzed as a function of mesh density. Minimal deviation in product yield was achieved with respect to mesh density for all reactor geometries and therefore the density of our mesh was considered sufficient for our analysis.

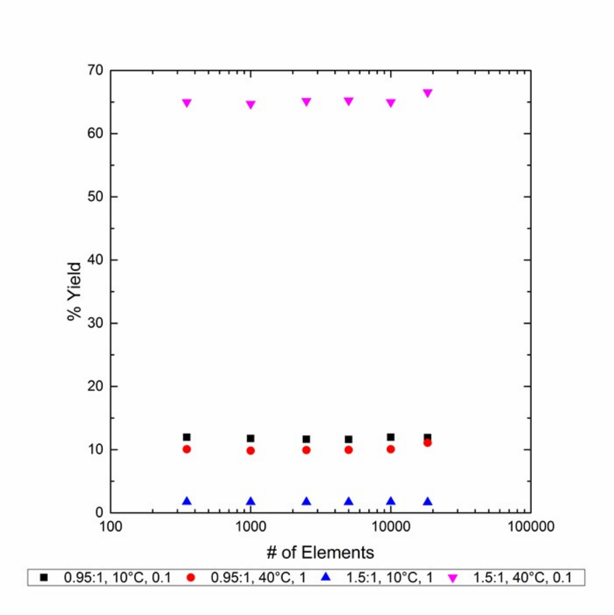

Figure S5. Product yield as a function of number of mesh elements for 1 m long, 1 mm ID reactor following DoE conditions given as molar ratio, temperature and volumetric flow rate in ml/min respectively.

Numerical Diffusion Analysis

Finite element based numerical simulations are prone to numerical diffusion which is an artifact of the computational approach which acts in addition to the specified diffusivity of the system. To evaluate the magnitude of this effect on our system, a numerical diffusion analysis was performed following the approach of Okuduc and Aral.[5] The effective diffusivity of the reactor was calculated from equations S5-S9 and compared with our specified diffusivity values.

$$D_{effective} = \frac{c_{inlet}^{2} - c_{outlet}^{2}}{2\tau(\nabla c)_{\forall}^{2}} \qquad \text{(S5)}$$

$$c_{inlet}^{2} = \frac{1}{Q}\int_{A} n \cdot uc^{2} dA \qquad \text{(S6)}$$

$$c_{outlet}^{2} = \frac{1}{Q}\int_{A} n \cdot uc^{2} dA \qquad \text{(S7)}$$

$$\nabla c_{\forall}^{2} = \frac{1}{\forall}\int_{\forall} (\nabla c) \cdot (\nabla c) d\forall \qquad \text{(S8)}$$

Additional simulations were carried out with the absence of diffusive terms in the mass transport equations to reveal the artificial diffusive response in the calculations. The numerical diffusivity was found to be low (2^{+} orders of magnitude smaller) with respect diffusion of the chemical species in the reactor and therefore its impact on our results was not considered significant.

Computational Fluid Dynamic Yield Results

A full factorial design of experiments study was simulated computationally encompassing the design space of the fractional, factorial, empirical, study addressed in the main text. Product yield as a function of reactor length was determined for all trials. Those matching the experimental study are shown in Figures S6-S9.

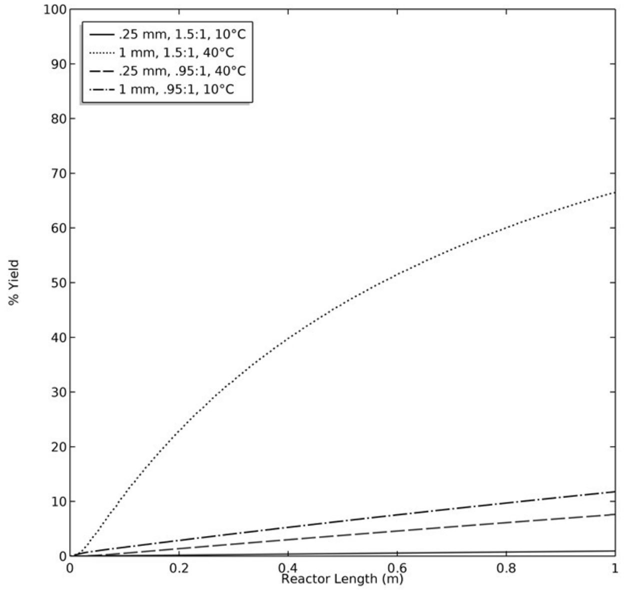

Figure S6. Product yield as a function of reactor length. Flow rate = 0.1 ml min^{-1}, reactor length = 1 m.

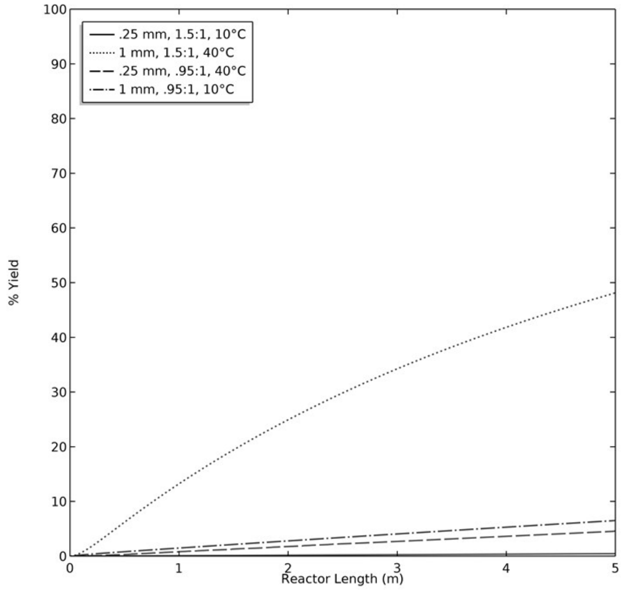

Figure S7. Product yield as a function of reactor length. Flow rate = 1 ml min^{-1}, reactor length = 5 m.

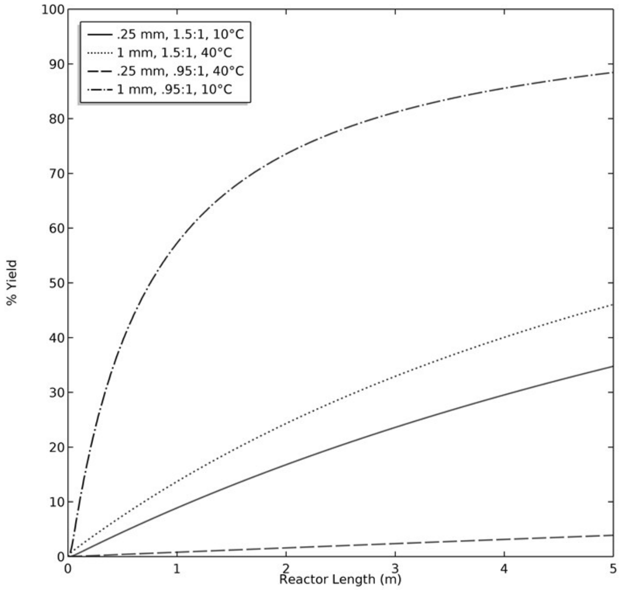

Figure S8. Product yield as a function of reactor length. Flow rate = 0.1 ml min^{-1}, reactor length = 5 m.

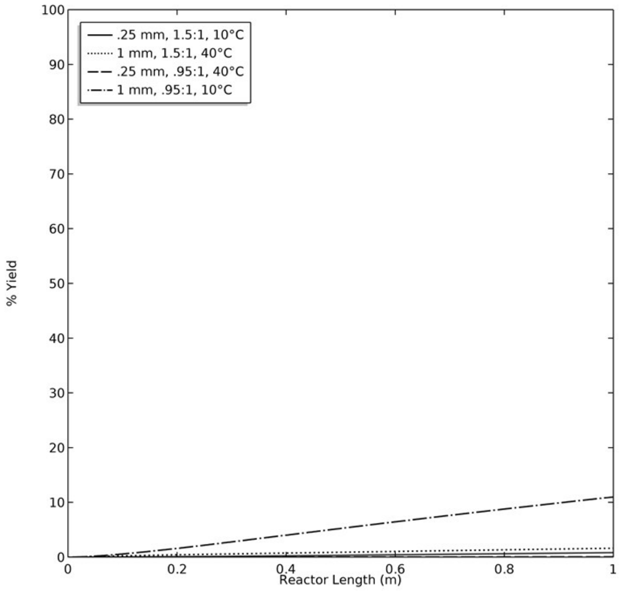

Figure S9. Product yield as a function of reactor length. Flow rate = 1 ml min^{-1}, reactor length = 1 m.

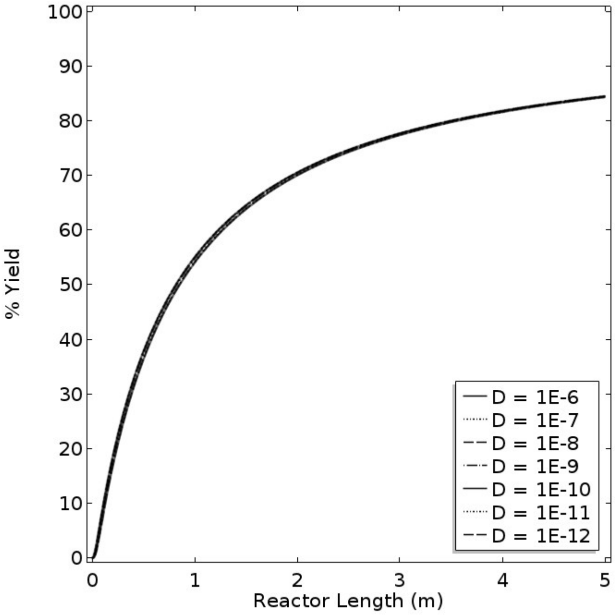

Figure S10. Product yield as a function of reactor length over sweep of species' diffusion coefficients. Reaction condition: 5 m reactor with 0.95:1 **DMF-DMA:M4MAA** molar ratio, 1 mm ID, and 0.1 ml min^{-1} flow rate at 40°C.

Design of Experiments Pareto Plot

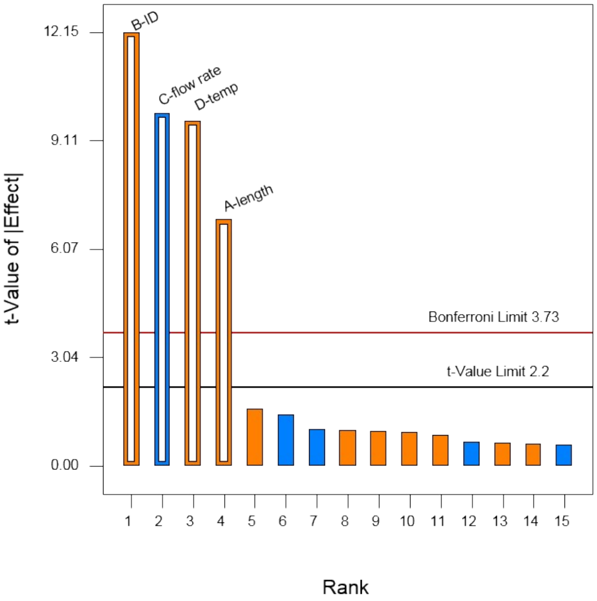

Figure S11. Pareto chart used in statistical analysis of CFD data

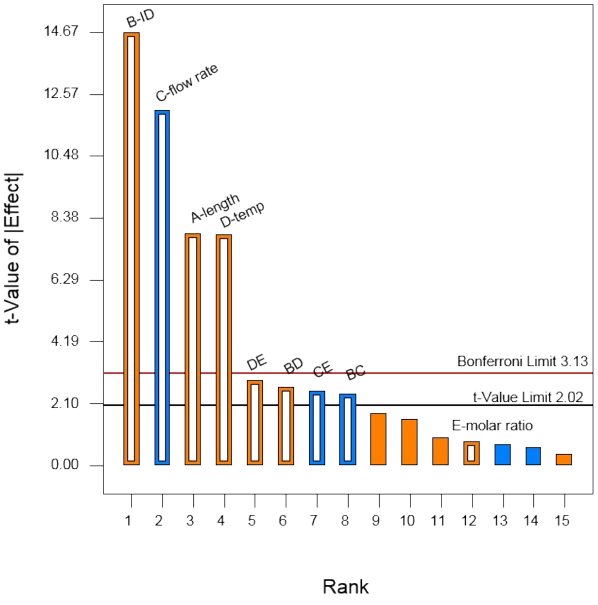

Figure S12. Pareto chart used in statistical analysis of experimental data

References

1 S. C. Rutan, A. de Juan and R. Tauler, in *Comprehensive Chemometrics*, eds. S. D. Brown, R. Tauler and B. Walczak, Elsevier, 2009, vol. 2, pp. 249–259.

2 A. de Juan, J. Jaumot and R. Tauler, *Anal. Methods*, 2014, **6**, 4964–4976.

3 K. -L Hsu, D. E. Kline and J. N. Tomlinson, *J. Appl. Polym. Sci.*, 1965, **9**, 3567–3574.

4 G. T. Furukawa, R. E. McCoskey and G. J. King, *J. Res. Natl. Bur. Stand.*, 1952, **49**, 273.

5 M. B. Okuducu and M. M. Aral, *Micromachines*, 2018, **9**, 1–28.
